# The emerging role of mechanical and topographical factors in the development and treatment of nervous system disorders: dark and light sides of the force

**DOI:** 10.1007/s43440-021-00315-2

**Published:** 2021-08-14

**Authors:** Natalia Bryniarska-Kubiak, Andrzej Kubiak, Małgorzata Lekka, Agnieszka Basta-Kaim

**Affiliations:** 1 Laboratory of Immunoendocrinology, Department of Experimental Neuroendocrinology, Maj Institute of Pharmacology, Polish Academy of Sciences, Smętna 12, 31-343 Kraków, Poland; 2 Department of Biophysical Microstructures, Institute of Nuclear Physics, Polish Academy of Sciences, 31342 Kraków, Poland

**Keywords:** Neuropharmacology, CNS diseases, Ischaemia, Mechanotransduction, Biomechanics, Elastic modulus

## Abstract

Nervous system diseases are the subject of intensive research due to their association with high mortality rates and their potential to cause irreversible disability. Most studies focus on targeting the biological factors related to disease pathogenesis, e.g. use of recombinant activator of plasminogen in the treatment of stroke. Nevertheless, multiple diseases such as Parkinson’s disease and Alzheimer’s disease still lack successful treatment. Recently, evidence has indicated that physical factors such as the mechanical properties of cells and tissue and topography play a crucial role in homeostasis as well as disease progression. This review aims to depict these factors’ roles in the progression of nervous system diseases and consequently discusses the possibility of new therapeutic approaches. The literature is reviewed to provide a deeper understanding of the roles played by physical factors in nervous system disease development to aid in the design of promising new treatment approaches.

**Graphic abstract:**

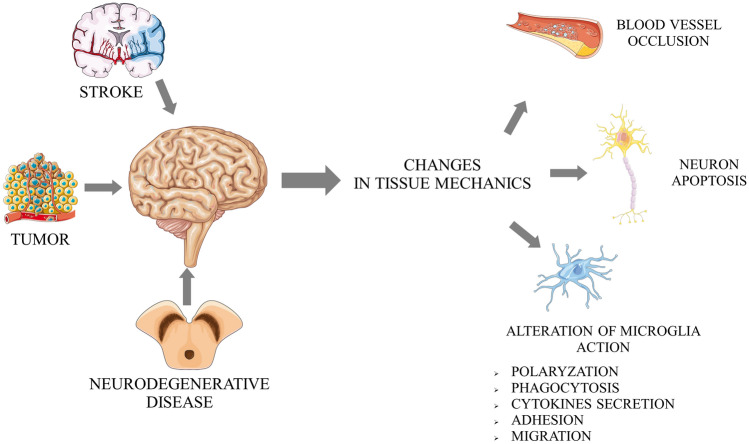

## Introduction

Central nervous system diseases frequently lead to severe disabilities or even death. This makes them a very important research topic, aiming to find new therapeutic strategies. To date, most therapeutic approaches treat various receptors responsible for intracellular signalling cascades as targets for molecules acting as drugs. Most of these strategies address biological alterations in cells as the leading cause of diseases [1]. Recently, far more evidence has shown that apart from biological changes in cells and tissue features, alterations in physical interactions inside biological systems lead to disease development and progression. Such pathological alterations include changes in tissue mechanics due to affected extracellular matrix (ECM) composition and functioning, reorganization of the cytoskeleton, and deregulation of cytoskeleton-associated protein expression. The clearest example of such a phenomenon is in heart and vascular diseases, where changes in shear stress that occur in atherosclerosis lead to pathological activation of endothelial cells. The importance of physical factors has also been recognized very well in cancer diseases at the molecular, cellular, and tissue levels. It has been shown that physical stimuli may reach the nucleus nearly 10^7^ times faster than chemical stimuli [2].

Consequently, changes in forces of interaction between cells and extracellular matrix affect multiple processes, such as their migratory properties, or modulate their drug resistance [3–6]. Cells are known to respond mechanically to anticancer treatment, changing their mechanical properties in a drug-dependent manner [7, 8]. Conversely, proper manipulation of the microenvironment's mechanical properties may increase the differentiation capability and regenerative potential of the cells [9–11]. Transmission of force into the biological signal is called mechanotransduction and is currently recognized as a new approach in understanding how diseases arise and propagate.

To date, less is known about the involvement of physical factors in the aetiology of central nervous system (CNS) diseases. A better understanding of the role of this phenomenon might lead to increasing the effectiveness of pharmacotherapy for CNS diseases.

## Mechanotransduction and mechanosensitivity

Mechanotransduction is a process of the conversion of mechanical stimuli into a biological signal. In principle, this phenomenon occurs when cellular receptors can be activated by extracellular force action or when extracellular forces are transduced via the cytoskeleton directly to the nucleus, affecting its organization and thus altering gene expression. One of the fundamental properties is the velocity of signal transduction. Comparing the transmission of mechanical stress along the cytoskeleton with chemical signalling, the velocity of stress transmission is approximately 30 m/s, while the velocity of small molecule diffusion is in the range of 1 μm/s. Thus, mechanical stimuli are at least 10^7^ times faster than chemical stimuli [2]. The physical signal might be transmitted directly to the cell nucleus by its propagation through various cellular cytoskeleton elements. The nucleus is connected to the cellular cytoskeleton by proteins of the linker of nucleoskeleton and cytoskeleton (LINC) complex. Members of this complex include two groups of SUN (for Sad1 and UNC-84) proteins connected to nuclear envelope laminins, and KASH family (Klarsicht, ANC-1, Syne homology) proteins interacting with cellular cytoskeleton elements such as microtubules, actin fibres, and intermediate filaments [12]. Such connections of the cell nucleus with all types of cytoskeleton filaments lead to the ability of the nucleus to feel force transmitted directly through those filaments. Consequently, mechanical stimuli lead to chromatin recombination, nuclear matrix distortion, transport changes through nuclear pores, and DNA melting. All these processes are then directly responsible for changes in gene expression [2].

On the other hand, one of the best-understood processes underlying mechanotransduction is based on integrins. These adhesive proteins play a crucial role in connecting the actin cytoskeleton to actin filaments. When mechanical stimuli are felt by integrin-based adhesions, they lead to changes in conformation and consequently in the functionality of proteins forming adhesions. In such cases, various signalling cascades may be activated and lead to intracellular signal transmission [13].

Finally, numerous recent studies have shown that changes in the mechanical properties of the cellular environment and the cells themselves affect processes such as stem cell differentiation [14, 15], immune response [16–18], the behaviour of disease-associated fibroblasts [19–21], cancer cell properties and their invasive potential [22–24], and cancer cell drug resistance [4, 7, 25, 26]. The significance of mechanical factors in various pathological conditions indicates that such factors should be considered when considering new therapeutic strategy development. As mentioned above, the most significant focus of such mechanical studies has been devoted to cancer, which intuitionally occurs because multiple processes such as invasion and metastasis are strongly related to purely physical phenomena. On the other hand, growing evidence indicates that central nervous system diseases strongly rely on pathological changes in their mechanical properties and physical interactions within nervous tissue. To better understand the significance of mechanical factors in brain tissue functioning, it is first important to understand how mechanical factors affect development and functioning during homeostasis.

### Impact of mechanical stimuli on neuronal development and neural cell differentiation

Embryonic development depends not only on changes in gene expression and biological signalling, but also on physical factors. Changes in the physical properties of developing embryos are crucial for breaking the symmetry of cellular divisions and differentiation of cells of further developmental stages into particular cell types. Phase transitions between solid-state and viscous or viscoelastic phases are currently attracting great interest. Such changes result from different cell activities—for instance, loosely attached and fast proliferating cells behave like a fluid, while strongly attached behave rather like viscoelastic or even elastic material. Consequently, such phase transitions between particular compartments in developing organisms lead to changes in how cells feel the force, thus affecting biological signal transduction [27]. Neuronal development is associated with the differentiation of ectodermal cells into a neural plate, giving rise to neural tubes and neural crests. Once the neural tube is formed from the neural plate, it is an “open” structure that tends to be closed during the neurulation process. Its closing is regulated by the action of the F-actin network, which plays a role in the interplay between active forces of apical cells responsible for gap closing and passive forces generated in the opposite direction by tissue tension [28]. Consequently, physical factors play a key role during neural crest cell migration and include the following mechanisms: actomyosin contraction, changes in tissue fluidity, confinement, contact inhibition of locomotion (CIL), and sensing stiffness of cell surroundings [29]. The stiffness of target tissue is a significant factor affecting the migratory capabilities of neural crest cells. Enteric neural crest cells are capable of spreading on substrates characterized by a very wide range of stiffness, with elastic moduli in the range of ~ 100 Pa–1 MPa. This ability is directly correlated with the stiffness of the tissue to which these cells migrate. Nevertheless, the stiffness of the 3D environment is an ultimate factor determining the migratory capabilities of enteric neural crest cells migrating with the highest velocity in soft gels with an elastic modulus of approximately 200 Pa. In contrast, for an elastic modulus of approximately 1 kPa, this migration is ultimately slower. Finally, for 3D gels with a stiffness of approximately 10 kPa, the cells are unable to migrate [30]. Any alteration of the proper functioning of biological and physical factors responsible for neural tissue-related development can lead to disease. For example, mouse Zic2^Ku/Ku^ mutants that possess functionally null ZIC2 alleles lead to the disappearance of actin cables, maintaining the proper functioning of the actin network. Once this robust actin structure is absent, ablation performed within the closing neural tube increases posterior neuropore widening and thus increases the chances of bifida development [28]. Thus, mechanical factors are important to study in disease modelling and treatment. Stem cells might be utilized to differentiate into daughter cells depending on stem cell type [31]. Among various stem cell types, neural stem cells (NSCs) are well known to give rise to neurons, oligodendrocytes, and astrocytes. While biological factors such as a proper composition of growth factors might direct NSCs into a particular lineage, mechanical factors alone can induce such targeting. Induction of 10% equibiaxial strain to murine and rat NSCs results in significant inhibition of their differentiation into oligodendrocytes, confirmed by staining of O4 oligodendrocyte markers. On the other hand, differentiation into neuronal lineage was elevated in the case of stretched cells, confirmed by an increased number of MAP2 (microtubule-associated protein 2)-positive cells on the stretched substrate. Importantly, decreases in oligodendrocyte lineage differentiation in response to stretching have been shown to rely on the interaction of NSC adhesion proteins with the ECM. Inhibition has been observed for cells cultured on laminin, while the use of fibronectin-coated substrates did not have the impact of stretching on oligodendrocyte lineage differentiation. Thus, it has been shown that the response to physical factors is also directly coupled with mechanotransductive pathways—in this case, the interaction between integrins and ECM proteins [32]. Shear stress and proper micropatterning of substrates for mesenchymal stem cell (MSC) neuronal differentiation have also played an essential role in obtaining the most effective differentiation protocol. A polydimethylsiloxane (PDMS) substrate with a groove/ridge width of 5 μm and shear stress of 0.1 kPa has been shown to simultaneously promote neuronal differentiation of MSC cells by increasing neurite length and inducing MAP2 expression as well as a higher calcium concentration [33]. Mechanical factors have also been reported to play a key role in neuron maturation and the formation of connections between particular neurons. A mechanical interaction has been shown to play a role in axon specification, growth, pathfinding, fasciculation, pruning, and synapse formation [34]. In *Xenopus* retinal ganglion cells, axons have been shown to prefer to grow in a soft environment, confirmed both in vivo and in vitro on elastic substrates. The ability of neurons to sense their surroundings has been shown to rely on the mechanosensitive protein [35]. Piezo1 is a mechanically activated ion channel Piezo protein. Importantly, it has been shown in NSCs to play a key role in neuronal differentiation of cells on stiff substrates. Modulation of Piezo1 functioning by the use of its inhibitor (GsMTx-4) resulted in preferential differentiation of NSCs into astrocytes and inhibition of neuronal differentiation [36]. Moreover, Piezo1 and Piezo2 mechanosensitive ion channels have been shown to play a crucial role in signal transmission in baroreceptor reflexes in neurons associated with the walls of blood vessels. Double knockout of these proteins in epibranchial placode-derived ganglia cells resulted in a significantly higher blood pressure increase in mice in which an increase in blood pressure was induced pharmacologically with the use of phenylephrine. Moreover, impairment of the proper functioning of the baroreceptor reflex results in higher variability in blood pressure than in control animals [37]. Such studies directly show that changes in the functioning of proteins involved in mechanical signal transduction in the nervous system may lead to pathological states and may be used as targets for therapeutic approaches. It should be emphasized that neuronal linage cells are responsible for homeostasis and pathological states in the brain, and as recently shown, immunocompetent cells play a key role in the proper functioning of the brain.

### Force-sensitive immune cells in the brain and peripheral nervous system

Inflammation and mechanical interactions within cells and tissues are closely related. Among others, atherosclerosis, in which chronic inflammation plays a key role, is observed to increase the rigidity of vessel walls. Lower arterial deformation leads to an increase in amplitude pressure (the difference between systolic and diastolic pressure that for the correct pressure is approximately 40 mmHg), resulting in the development of difficult-to-treat isolated hypertension [38, 39]. Similarly, in the case of cartilage, mechanical changes resulting from the action of proinflammatory cytokines lead to impairment of cartilage function [40].

**Fig. 1:**
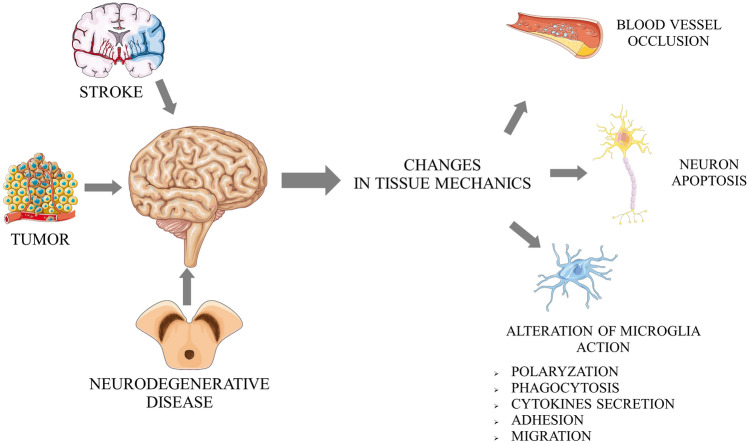
Schematic presentation of mechanical cues in nervous disease development and progression. Not only does mechanically based traumatic brain injury affect brain mechanics, but also multiple types of diseases (stroke, brain tumours, or neurodegenerative diseases) induce changes in brain tissue mechanics and nanotopography. These physical alterations are then transduced into a biological signal that leads to additional negative effects in multiple cases. The figure is the original work of authors using Servier Medical Art graphics licenced under a Creative Common Attribution 3.0 Generic Licence (http://smart.ser-vier.com) based on reference nos. [43, 66, 86, 87, 92]

Changes in the mechanical properties of tissues have a significant impact on functioning immune system cells. In the case of antigen-presenting dendritic cells, changes in the mechanical properties of the environment alter the ability of these cells to internalize antigens and affect the expression of proteins (CD83 and CCR7) responsible for chemokine-dependent migration. It also seems that the changes resulting from disease states such as tissue fibrosis or ECM rigidity can change the way the immune system responds [41]. Similarly, in macrophages, substrate rigidity affects the degree of their activation under the influence of the activator of the immune system, lipopolysaccharide. Using polyethylene oxide gels for the stiffest substrate (840 kPa), the expression of genes for proinflammatory cytokines (IL-1β and IL-6) was significantly higher than for substrates with lower stiffness (130 and 240 kPa) [16]. A similar trend was demonstrated for macrophages grown on polyacrylamide gels, where the increase in expression of proinflammatory cytokines (IL-1β, TNF-α, and IL-6) correlated with an increase in gel stiffness [17]. Substrate flexibility has a direct impact on macrophage behaviour. Changes in the topography of the substrate may also affect their activation and adhesive capacity [42]. Very often, an increase in stiffness of the environment leads to the activation of immune cells [16, 17]. A similar effect was observed for microglia cells. Increased substrate rigidity led to changes in their morphology (more cells with the activated microglial phenotype) and significantly increased inflammatory cytokine expression. The authors emphasize that such “mechanosensitivity” of microglia is responsible for their activation in the case of brain electrode transplantation (Fig. 1) [43].

## The nature of the force in the CNS

### Methodologies used in studies on cell and tissue mechanics

While multiple techniques have been applied to investigate the biochemical and genetic features of biological samples, in recent years, multiple methods have been developed to investigate their physical properties. Magnetic resonance elastography has been widely used to study the mechanical properties of living tissues. This method allows for the determination of the shear moduli of investigated tissue. This is ultimately useful while thinking about nervous tissue because of these tissues’ virtual inaccessibility for biopsy or another invasive measurement. On the other hand, its disadvantage is low resolution compared to more precise techniques [44]. Among these, Brillouin spectroscopy serves as a very promising approach for the noninvasive characterization of biological samples with a 3D resolution, allowing for far more elasticity mapping of the cellular compartment compared with the use of the aforementioned MR elastography [45]. Recently, this technique has been applied to investigate changes in the mechanical properties of murine embryos during cranial neural tube closure, showing significant differences in mechanical properties between different tissue components as well as the same tissue components depending on development stage [46]. On the other hand, experimental methods such as in vitro cell culture of single cells embedded in hydrogels mimicking the physical properties of ECM require different approaches. Among these, rheological measurements are very useful for characterizing hydrogels, polymers for cell culture, or tissue samples [47, 48]. On the other hand, for a proper characterization of mechanical forces exerted by cells on their surroundings and the parallel stiffness of cells and their particular compartments, traction force microscopy and atomic force microscopy are recognized as gold standards and thus will be described more precisely in the following section.

#### Traction force microscopy

One of the methods that allows measuring the forces generated by cells in their surroundings is traction force microscopy (TFM). This method measures how the cell deforms (*d*), a flexible substrate with known mechanical properties (known constant elasticity, *k*). By applying Hook’s law, i.e. *F* = *k* × *d*, it is possible to calculate the traction force (*F*) generated by a cell in a given place. In a classic approach, traction force microscopy measures substrate deformation by tracking the position of fluorescently labelled beads incorporated into a gel substrate. The deformation is obtained by comparing two recorded images showing fluorescent beads with and without cells. Knowing the deformations resulting from the action of forces generated by the cell and the mechanical properties of the employed gel substrate, the forces can be calculated at each bead position [49, 50]. In breast cancer, MCF7 tumour cells have been shown to generate greater traction forces than epithelial cell breasts of the MCF10A line [51]. Moreover, for breast, prostate, and lung cancers, metastatic cells generate larger traction forces than nonmetastatic cells before metastasis [52]. TFM has been applied to describe the forces generated by cells during mitotic divisions [53] and to demonstrate the formation of systolic centres during neuronal migration [54]. Traction forces have been measured for Schwann cells, which play a key role in repairing the peripheral nervous system. The magnitude of traction forces generated by these cells rises with increasing stiffness of the substrate in which they were cultured [55]. A similar trend has recently been observed for microglia. It has been found that these cells possess durotaxis, i.e. the ability to migrate along a rigidity gradient towards a stiffer substrate. This process was further enhanced by activating a nonspecific microglial activator of the immune system, lipopolysaccharide, and changes in traction forces correlated with a greater glial cell migration rate [56]. This work shows the mechanical sensitivity of microglial cells and their tendency to migrate to a stiffer environment where microglia are more strongly activated than in an environment with lower rigidity [43, 56]. Importantly, traction force microscopy has been applied in studies of growing axons. Polackwich R. et al. showed how axon growth cones generate a stress field around growing axons. TFM allowed observation of the dynamic fluctuation of forces generated by growing axons on their surroundings[57]. Traction force microscopy has also been used to study differences in growth cone behaviour between central nervous system neurons (hippocampal) and the peripheral nervous system (dorsal root ganglion—DRG). DRG neurons are characterized by stiffness-dependent neurite outgrowth and generate higher forces than hippocampal neurons, and neurite outgrowth is independent of substrate stiffness [58]. Finally, the use of *Aplysia* bag neurons allowed to describe how traction forces contribute to growth cone advancement. In detail, the authors show that after relaxation of one of the sides of the growth cone, forces are redistributed to the opposite side. As a result, the central doimain of the growth cone is pulled into the region of higher tractions, followed by proportional redistribution of traction forces across the whole growth cone. Consequently, neurites are elongated, and in the next step, a similar event occurs but in the opposite order [59].

#### Atomic force microscopy

To study the mechanical properties of soft samples such as cells, hydrogels, and tissues, atomic force microscopy (AFM) is widely used [60, 61]. AFM relies on scanning a sample with a cantilever possessing a probe on its end. This technique was initially developed to perform nanoscale object topography measurements; nevertheless, progress in AFM application has led to its use in the mechanical characterization of investigated samples. Knowing the mechanical and geometrical properties of the cantilever and AFM probe, it is possible to measure the sample topography and force of interaction between the sample and cantilever [62]. Such force can be recalculated to determine the mechanical properties of a measured sample using the Hertz–Sneddon model of indentation [63]. As a result, the Young’s modulus of the investigated sample is obtained, describing the sample elasticity. A larger Young’s modulus denotes greater stiffness and consequently lower deformability. Initially, numerous experiments were focused on cancer biology, indicating the general conclusion that cancer cells are softer than their healthy counterparts, while for tissue, the opposite trend was observed [61, 64]. Recently, AFM has been utilized to show that prostate cancer cells stiffen after treatment with chemotherapeutic agents such as cisplatin or microtubule interacting drugs [25, 65]. Studies in this area reveal the significance of mechanical changes in cancer progression and provide a more in-depth explanation of pathogenesis as well as cellular response to treatment. In the case of cancer, tissue stiffening is associated with tissue fibrosis [66]. Such findings are extremely important because it has been shown that stiffening of the cell culture environment can induce epithelial–mesenchymal transition and induce chemoresistance in cancer cells and thus worsen the prognosis [67, 68]. Findings addressing cancer biology reveal the significance of AFM measurements in both discovering various diseases’ pathogenesis and their potential treatment. In 2004, McNally and Borgens applied AFM to investigate changes in the morphology of neurons in standard culture and during the induction of their death. AFM measurements allow high-resolution topography of growing neurites and prominent cell bodies. Importantly, in this study, neuronal death was induced by breaking the cell membrane using an AFM tip. As a result, degradation of neurons was reported not only at qualitive levels (degraded cell bodies and malformed cytoplasm in topography images), but also at quantitative levels (significant decreases in cell body volumes and increases in volumes of cytoplasm) [69]. High-speed atomic force microscopy has been used to investigate real-time changes in the dynamics of dendrites in rat hippocampal neurons. Importantly, this method possesses good resolution, allowing us to observe signs of endocytosis and rapid movements of dendrite extension [70]. In 2012, Spedden et al. performed combined AFM elasticity mapping and fluorescence microscopy of three neuronal lines: embryonic rat cortical neurons, embryonic chick dorsal root ganglion and P-19 cell line-derived neurons. In the first step, these three cell lines were compared in terms of their mean elasti moduli. Both P-19-derived neurons and rat cortical neurons were characterized by similar mechanical properties—mean elastic moduli between 0.2 and 0.4 kPa. Dorsal root ganglion neurons were characterized by a significantly higher mean elastic modulus of 0.9 kPa. Further parallel elasticity mapping and fluorescent imaging of cortical neurons showed that the mechanical properties of neurons rely mostly on microtubules, but not the actin cytoskeleton [71]. Recently, physical markers of neuronal damage induced by oxidative stress were discovered with the use of AFM. Induction of oxidative stress by H_2_O_2_ results in malformation of neuronal morphology and disappearance of protrusions in the cell membrane, characteristic of control neuronal cells. Interestingly, the use of quercetin, an antioxidative agent, results in decrease in the magnitude of changes in the morphological properties of neurons. Finally,, treatment of neurons with H_2_O_2_ ultimately resulted in an increase in the mean elastic moduli of neurons from 2.4 ± 0.2 kPa in the control to 14.7 ± 0.5 kPa in H_2_O_2_ cells. Similar to observations derived from AFM-based analysis of neuronal topography, treatment of H_2_O_2_-stressed cells with quercetin resulted in an intermediate elastic modulus value of 8.7 ± 0.7 kPa. Finally, these data were strongly supported by biological data in which the use of quercetin led to a significant decrease in H_2_O_2-_induced apoptosis. Such observations allow the authors to conclude that AFM might be used as a promising tool to investigate physical markers of the cell response to both stress and treatment [72].

### Mechanical interactions in stroke and neurodegenerative diseases

**Fig. 2:**
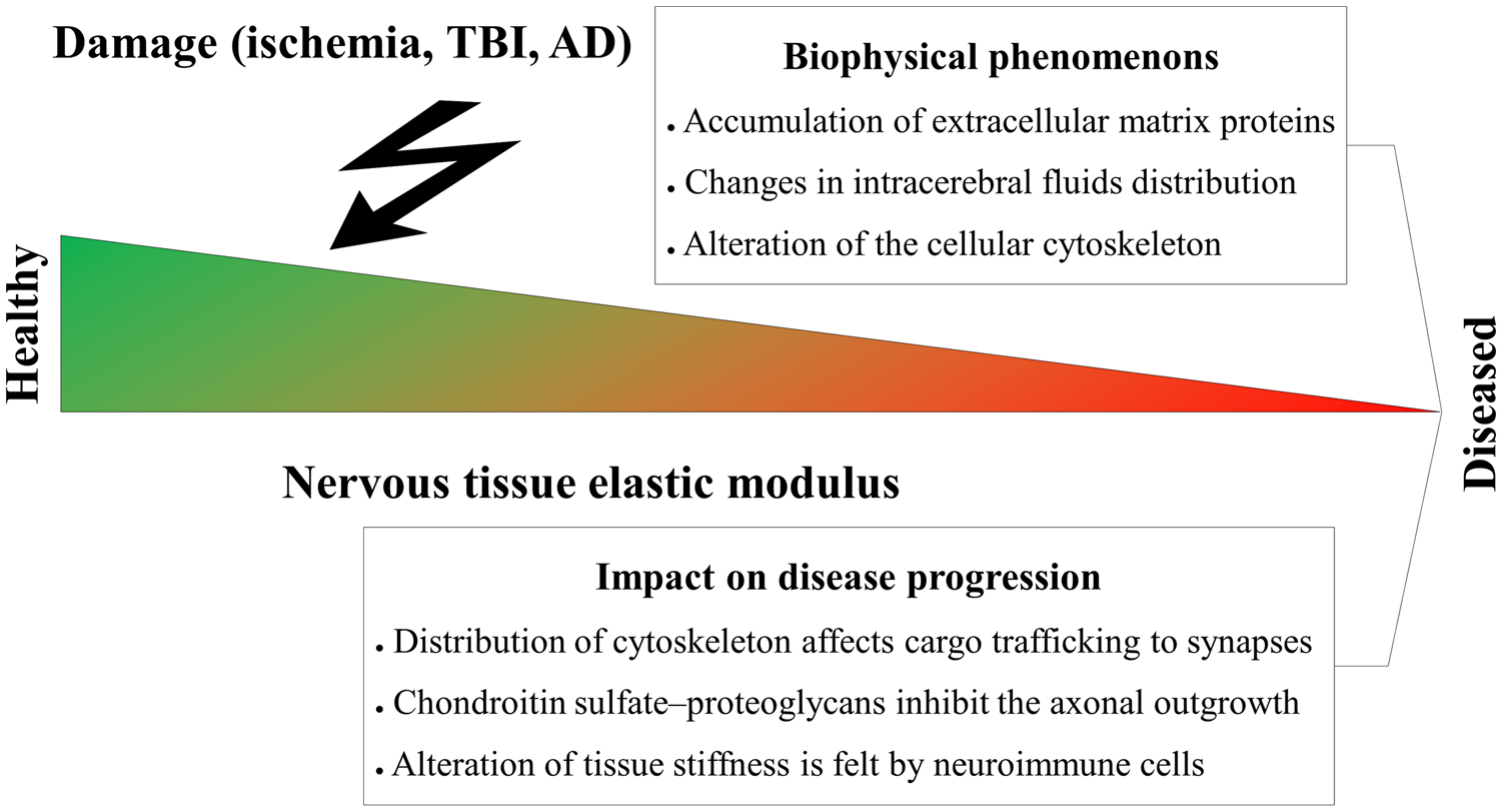
Role of pathological softening of nervous tissue in disease progression and inhibition of tissue regeneration. Based on references no.: [84–87, 92]

In 2019, Seano et al. showed that nodular glioblastoma tumours destroy surrounding neurons by generating mechanical forces. Additionally, solid tumour growth forces led to decreased vessel diameters in the brain areas adjacent to the tumour (Fig. 1) [73]. Considering the physical mechanism of damage to nerve tissue due to the forces generated by a solid tumour, the search for a similar phenomenon in ischaemic stroke is an important issue. Cells accumulate within the affected nerve tissue after activation of the immune system. This may lead to a similar generation of solid stress in an ischaemic region as in nodular glioma. Ischaemic stroke leads to several pathological changes in brain tissue. Two basic mechanisms leading to brain damage are induction of neuronal death resulting from a lack of oxygen and nutrients, and subsequent damage to the nervous tissue resulting from reoxygenation and inflammation in the stroke area [74, 75]. The first of the above mechanisms results from neurons' high energy demand, making them extremely vulnerable to limited access to oxygen and glucose [76]. The second is associated with an increased concentration of reactive oxygen species (ROS), a consequence of tissue reperfusion and the influx of immune cells [77, 78]. As described above, any alterations in the mechanical properties of the cell environment potentially change the biological response of immune cells. Among all immune cells, the role of microglia in the course of stroke is the most commonly investigated. Microglia are among the brain-specific cells of the immune system. Activation occurs under the influence of various factors disrupting tissue homeostasis. As a result, microglial cells secrete several cytokines, growth factors, and chemokines. The proportions of secreted factors depend on the type of disease [79]. Ischaemic stroke is one of the pathological conditions during which microglia are activated. Its degree is proportional to the severity of the stroke. After ischaemic stroke, microglial cells undergo classic processes, including morphological transformation, proliferation, and polarization [80]. Under the influence of chemotactic factors, microglial cells migrate to damaged areas where they phagocytize dead cells. Once on site, depending on polarization, microglial cells can contribute to progressive tissue damage or regeneration [81]. Two main phenotypes of microglia affect polarization: proinflammatory (also called M1-like) and anti-inflammatory (often called M2-like). Activated M1 microglia secrete a series of proinflammatory cytokines, such as TNFα, IL-23, IL-1β or IL-12, one of the consequences of inflammation of the brain. A potential strategy for treating the effects of ischaemic stroke is the modulation of receptors of selected chemokines present on microglia [82]. These include CCL2, CXCL12 receptors, and ultimately fractalkine receptor (CX3CR1), expressed only on microglia but not neurons and astrocytes and thus a frequent subject of interest in such investigations [83–86]. Thanks to a specific location, the CX3CL1–CX3CR1 system plays an important role in the interaction between neurons and microglia, thus constituting a system of communication between these cells [87–89]. For example, the interactions between CX3CL1 and CX3CR1 regulate the neurogenesis processes, create neural networks and synaptic connections, and regulate the microglial phenotype. It should not be forgotten that in parallel to changes in the biological properties of immune system cells, their activation affects the secretion of cytokines and chemokines and the activation of their receptors and biomechanical properties [90]. Thus, questions about changes in the mechanical response of those cells and the impact of physical signals from their niche are key in considering therapeutic approaches. In animal models, the formation of glial scars after stroke is well described. Recently, the formation of such pathological changes in nervous tissue has been observed in humans. Such a structure is enriched in activated astrocytes and ED1-positive microglia and the accumulation of chondroitin sulphate proteoglycans (CSPGs), known to inhibit the regeneration of neuronal tissue (Figs. 1, 2) [91].

Importantly, in animal models, it has been shown that glial scars are significantly softer than healthy tissue. This observation is ultimately interesting because scars are usually pathologically stiffer than healthy tissues in most peripheral tissues. Softening of a scar is correlated with increased expression of extracellular and cytoskeleton-associated proteins such as laminin, vimentin, GFAP (glial fibrillary acidic protein), and collagen IV. Importantly, both the expression of proteins and a drop in scar stiffness decreased over time, but remained significantly altered. The significance of these changes also depends on the type of neuronal tissue that undergoes injury. While in the case of both grey and white matter, a drop in elastic modulus was very significant inside the lesion, in the case of the region outside the lesion such significance was preserved only for grey matter, while for white matter, there was no significant difference compared to the sham groups. Such mechanical alteration may thus inhibit the tissue's ability to be regenerated on different levels (Fig. 2) [92]. In human NSCs, stiff substrates are responsible for promoting neuronal differentiation of NSCs [36]. Thus, tissue softening after brain injury can be treated as one of the additional negative factors responsible for poor neuronal tissue regeneration. Such softening has been observed directly in the thromboembolic middle cerebral artery occlusion model of stroke. A decrease in the Young’s modulus of brain tissue has been observed when comparing the ischaemic site to the control site. What is more the ischaemic zone has been reported to be significantly softer than the border zone (Fig. 2) [93]. Such findings agree with data obtained from ultrasound elastography imaging of mouse brains. Here, 24 h after the MCAO (middle cerebral artery occlusion) procedure, significant decreases in the shear modulus of the ipsilateral hemisphere were observed with parallel significant increases in the shear modulus of contralateral hemisphere. These differences tended to lose significance 72 h after MCAO procedures. Significant differences between ipsilateral and contralateral hemispheres were also observed 72 h after MCAO as well as in control animals. The softening of the ipsilateral hemisphere and parallel stiffening of contralateral hemispheres were attributed to ipsilateral oedema progression—thus to the macroscopic process (Fig. 2) [94]. Mechanical changes in the brain affected by ischaemia seem to result from both changes at the scale of proteins and cellular behaviour and macroscopic changes in the behaviour of fluids. In both cases, cells are capable of detecting changes in force exerted upon them, and thus any deviation from the physiological mechanical state of tissue is capable of affecting the proper behaviour of immune cells and tissue regeneration. Mechanical changes may also be treated as a predictive factor in stroke prevention. It has been shown that platelets from smokers, well known to have a significantly higher risk of stroke, are softer (44.65 ± 0.23 MPa) than platelets of control patients without significant risk factors (57.56 ± 0.23 MPa), while platelets derived from patients suffering stroke had the lowest elastic modulus (34.91 ± 0.16 MPa). Such findings clearly show that mechanical parameters of relatively easily accessible cells, platelets, might serve as a prediction marker of severe neurological disease, i.e. stroke [95]. On the other hand, with shear wave elastography, it has already been shown that the longitudinal elasticity modulus is significantly elevated in patients suffering stroke [96]. It should be pointed out that ultrasound shear wave elastography has been limited to the assessment of stroke. Li-juan et al. observed that this method allows us to investigate pathological changes in muscle stiffness that occur during neurodegenerative disease progression. Significant stiffening of the investigated muscles was observed for patients with robust symptomatic and mildly symptomatic arms with elastic moduli of 59.94 ± 20.91 kPa and 47.77 ± 24.00 kPa, respectively. For control patients, elastic moduli for corresponding muscles were ultimately lower by 24.28 ± 5.09 kPa. In the same study, the authors noticed a linear correlation between the elastic moduli of muscle and the unified Parkinson's disease rating scale. Patients with the stiffest muscle also acquired the highest score on the scale, where a higher score denotes a larger impairment of motion [97]. Since multiple studies have shown the importance of mechanical alterations in nervous tissue, mechanical markers have been proposed as diagnostic tools in neurological disease. Recently, Pyka-Fościak et al. showed very promising results regarding the use of changes in Young’s moduli of the spinal cord as a very early marker of the development of multiple sclerosis. Significant stiffening of white matter has been observed in the onset phase of disease, while at that stage standard pathological assessments (inflammation degree and demyelination) have been reported only at low levels [98]. Recently, changes in the mechanical properties of particular brain structures have been shown to be an important marker for Alzheimer’s disease (AD) diagnostics. Magnetic resonance elastography (MRE) performed on patients suffering AD showed that they have significantly decreased shear moduli for the cerebrum, white matter, cerebral cortex and subcortical grey matter (Figs. 1,  2) [99]. Thus, alterations in the physical properties of nervous tissue have been widely shown to play important roles in pathogenesis by affecting neuroinflammation as well as axonal outgrowth. Moreover, recent studies provide evidence that these mechanical changes may be used as new diagnostic markers of disease. Another important possibility arises—a proper use of this knowledge to project new therapies for neurological disease.

## A new hope: can we use force to treat neurological diseases?

### Small molecules targeting mechanotransductive pathways

**Fig. 3:**
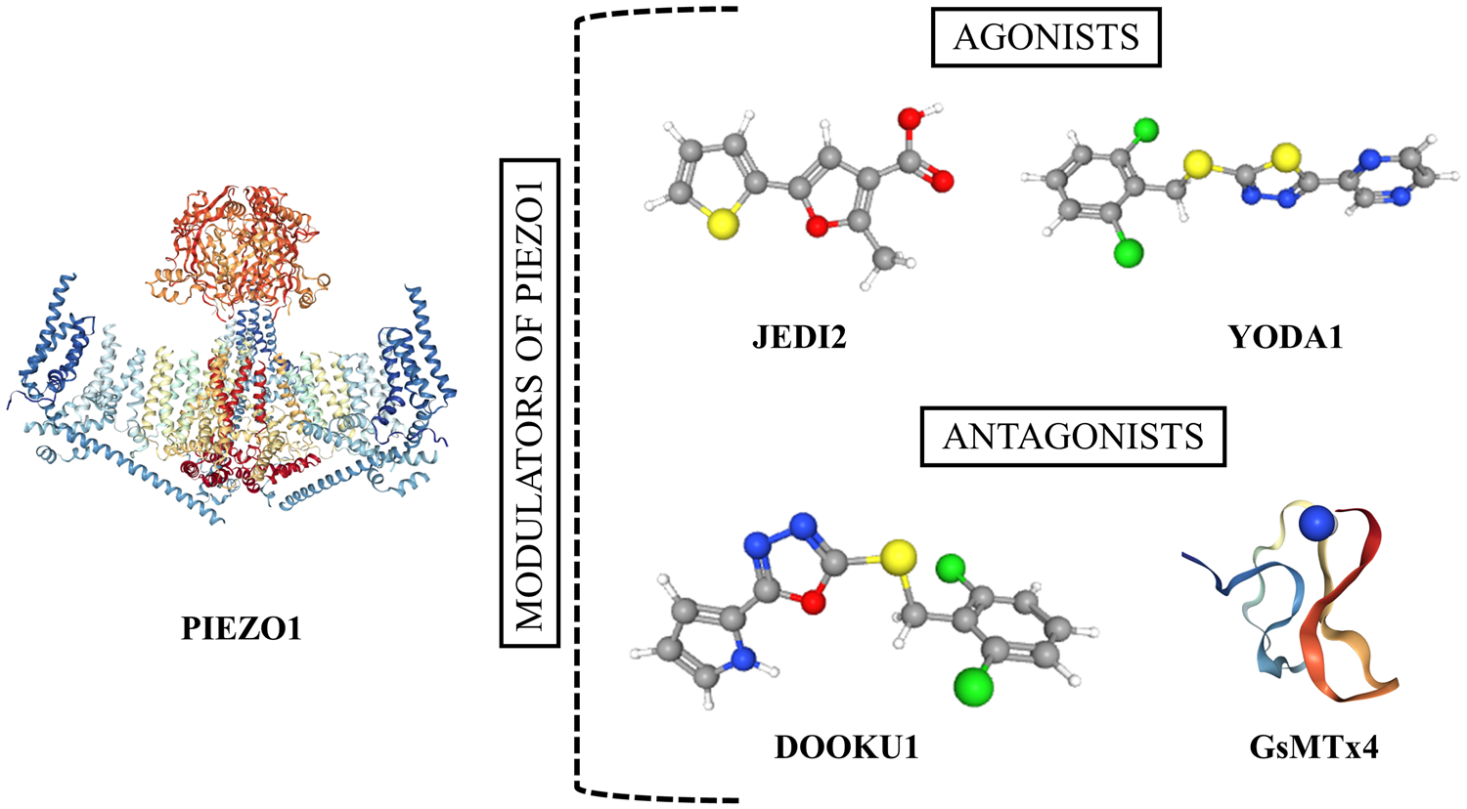
Among mechanosensitive proteins, PIEZO1 (PDB: 6BPZ) acts as a very promising target for potential therapies of nervous tissue diseases. Expression of its inhibitor GsMTx4 (PDB: 1 LU8) is elevated after brain tissue injury and is negatively correlated with axon outgrowth potential. On the other hand, JED2 (CID 2796026) and YODA1 (CID 2746822), selective agonists of the PIEZO1 protein and its selective antagonist DOOKU1 (CID 137321150), have recently been developed and could be used for the treatment of nervous system diseases. Images of molecules were obtained from PDB and PubChem. Their particular IDs are provided above. Based on references no.: [93–95]

Pathways dependent on mechanical interactions play a key role in developing and progressing multiple neurological diseases. They are promising candidates for drugs targeting particular neurological diseases. In Piezo1, its agonists Yoda1 and Jedi1/2 have already been developed together with Dooku1, an antagonist of the Piezo1 receptor. The application of these small molecules has been shown to exhibit promising therapeutic effects in migraine pain and neonatal diabetes treatment (Fig. 3; Table 1) [100–102].

**Table 1:** Proteins related to mechanotransduction and mechanosensitivity with their modulators

Protein	Modulators	Application	References
Piezo1	Yoda1, Jedi1/2, Dooku1	Migraine pain	[93–95]
αVβ3 and αVβ5 integrin	Cilengitide	Glioma	[97]
α4β1 integrin	Natalizumab	Alzheimer’s disease, traumatic brain injury	[101–103]
Focal adhesion kinases (FAK)	PF-573228	Glioblastoma	[106]
Rho GTPase	Rhosin	Neural differentiation	[107]
Rho-associated protein kinase (ROCK)	Fasudil	Alzheimer’s disease, stroke	[108–112]

A potential therapeutic application of these modulators is provided based on the relationship between a disease and a protein involved in its pathogenesis. Some of these modulators have been tested in preclinical models or used in clinical trials of nervous tissue diseases

Recently, PIEZO1 was reported as a potential prognostic marker of glioma. Genetic analysis of 1633 glioma samples revealed that higher expression of PIEZO1 was correlated with worse prognoses for patients. In glioma, PIEZO1 plays a key role in pathways regulating the expression of proteins associated with ECM and ECM remodelling, thus promoting disease progression and malignancy [103]. Among recent developments, the use of Dooku1 may be an interesting strategy against glioma variants with high expression of PIEZO1. Such a prospective approach is supported by other recent studies emphasising the contribution of PIEZO1 to the development of glioma malignancy [103] and elevated expression of PIEZO1 in peritumoural brain oedema, associated with worse prognoses in glioma patients [104]. Nevertheless, the potential role of PIEZO1 is not limited to brain cancer treatment. Ultrasonic stimulation of the brain is proposed as a noninvasive method to stimulate neurons, with very promising potential in the treatment of diseases such as stroke, Parkinson’s disease or chronic pain [105]. Recently, a crucial role for PIEZO1 in the response to ultrasonic stimulation of murine neurons was described by Qiu et al. The authors of this work showed that ultrasound is capable of activating PIEZO1 channels. Importantly, both inhibition of PIEZO1 by GsMTx4 and knockdown of PIEZO1 resulted in inhibition of the downstream effects of PIEZO1 activation (i.e. elevated expression of nuclear c-Fos) [106]. As mentioned in the previous section, integrins are crucial components of adhesive proteins responsible for mechanosensing and mechanotransduction. Cyclic peptides EMD 121974, EMD 85189, and EMD 66203, selective inhibitors of αVβ3 and αVβ5 integrins, have been shown to successfully inhibit angiogenesis by inhibiting endothelial cell invasive potential as well as their capability to differentiate upon stimulation with growth factors EGF-A and FGF-2 [107]. In the case of brain tumours, integrins are widely considered a target of anticancer therapies. Cilengitide—pentapeptide, which targets αV integrins, has been under evaluation in clinical trials up to phase III for treatment of glioma (Table 1) [108]. Preclinical studies on integrins targeting protein-based drugs, tumstatin and endostatin have shown that they inhibit glioblastoma growth in an animal model by 83% when used in combined therapy. While those agents were used separately, they inhibited tumour growth by approximately 50–58% [109]. Targeting α6 integrin in primary glioblastoma cell lines has increased cell susceptibility to radiotherapy [110]. Cancer is a disease that might be treated by targeting particular integrin subunits. As a good example, natalizumab (anti-α4 antibody) has been accepted in clinical practice in the USA since 2004 for multiple sclerosis treatment. Its action relies on the inhibition of α4β1–vascular cell adhesion molecule interactions. The aforementioned pathological inflammation responsible for MS progression is slowed (Table 1) [111, 112]. α4β1 integrin has been shown to be an attractive target for therapy for Alzheimer’s disease. α4β1 integrin targeting in 3xTg-AD mice using an anti-α4β1 antibody resulted in a significant decrease in AD severity by reducing disease-associated pathological processes, i.e. microgliosis or tau hyperphosphorylation and improvement of cognitive functions in animals [113]. A similar approach, i.e. inhibition of leukocyte infiltration by targeting α4β1 integrin, has also been applied in an animal model of traumatic brain injury. The use of monoclonal antibodies targeting α4β1 integrin led to decreased infiltration of immune cells into the spinal cord after injury and indicated improvement of neurological function in rodents after treatment [114]. As mentioned above, integrins play a key role in the formation of focal adhesion. During the formation of these structures, integrins interact with multiple types of proteins, including focal adhesion kinases (FAKs), which are also important in mechanotransduction pathways. FAK has been shown to transduce mechanical signals, crucial for the proper process of development [115]. Its modulation by the FAK14 inhibitor leads to increased neurogenesis in adult male C57BL/6 mice [116]. Additionally, such modulation might be considered positive when related to the awakening of endogenous repair mechanisms in the brain and negative when considering cancer treatments. A small molecule (PF-573228) FAK inhibitor has been successfully used to inhibit glioblastoma growth in vitro in both 2D and spheroid models of this cancer (Table 1) [117]. Other proteins associated with cytoskeleton and cell movements have been shown to serve as an attractive target for modulation in neurological diseases. Modulation of Rho GTPase with the use of its selective inhibitor rhosin leads to increased neurite outgrowth in PC-12 neuroblastic cells and thus might serve as a potential positive modulator of neuronal differentiation (Table 1) [118]. What is more, use of Rho-associated protein kinase (ROCK) inhibitor—fasudil—has shown promise in treating Alzheimer’s disease.

In the preclinical model of AD, its use results in inhibition of neuroinflammation due to reduced activation of microglia and astrocytes (Table 1) [119]. In another study by Ding et al., fasudil was shown to promote neural stem cell action in a hypoxia–reperfusion rodent model. Mice that underwent hypoxic conditions were injected with the drug. Neurogenesis in fasudil-treated animals was significantly increased. NSCs were mobilized from the subventricular zone, and their proliferation was significantly increased. Therefore, a significantly higher number of cholinergic progenitor cells were generated in fasudil-treated mice 7 days after hypoxic treatment (Table 1). Moreover, the drug stimulated astrocytes to express the cytokine granulocyte colony-stimulating factor (G-CSF) [120]. Corresponding results have been shown in vitro where treatment of murine cerebellum-derived C17.2 cells with fasudil led to enhanced neurite outgrowth and C17.2 cell differentiation [121]. In a study performed on Wistar rats with an intracerebral haemorrhage model, fasudil was shown to decrease inflammation and brain oedema as well as neuronal loss (Table 1) [122]. Finally, fasudil has been shown to improve both neurological functions and clinical outcomes in a phase IIb multicentre, double-blind clinical trial for patients suffering stroke, where it was administered within 48 h of stroke onset. Such promising results indicate that fasudil might be a very potent drug in the treatment of stroke, while its administration is not limited to a very narrow therapeutic window, as in the case of anticoagulant therapy (Table 1) [123].

### Mechanical models of brain and biomaterials of defined topography to gain a better understanding CNS cell functioning

Mechanical factors and mechanotransductive pathways represent emerging factors in considering neurological disease treatment, not only pertaining to use of particular drugs but also treating these factors as a target. The mechanical and nanotopographical features of the cellular environment serve as a key factor affecting the proper functioning of the nervous system. They might be used to enhance therapeutic outcomes—for instance, by increasing stem cell proliferation and differentiation. The brain is considered to be the softest tissue in mammals. Its mechanical properties result from both its cellular composition (neurons characterized by cell bodies and long axons, astrocytes, microglia, and endothelial cells) and brain-unique extracellular matrix (i.e. type IV collagen, hyaluronan, heparan sulfate proteoglycan, or chondroitin sulfate proteoglycan). During disease, their composition is affected, leading to alterations in nervous tissue mechanics as well as nanotopographical features [124]. Such changes are felt by cells, are active participants in disease progression, and might affect treatment. Recently, astrocytes, microglia, and corresponding PNS Schwann cells have been become well known to be playing an important role in the regeneration of the nervous system after injury. While these cells have been reported to exhibit both anti-inflammatory and pro-regenerative phenotypes as well as proinflammatory and regeneration-inhibiting phenotypes, it is important to identify factors that might direct glial cells into a pro-regenerative phenotype. High molecular weight hyaluronan and fibrinogen-coated aligned collagen fibres have been shown to decrease axon regeneration expression, inhibiting chondroitin sulfate proteoglycan by astrocytes [125]. Primary rat astrocytes have been shown to respond differentially to surface nanotopography. Astrocytes cultured on pitted and divoted, but not smooth, electrospun poly-l-lactic acid fibres have been shown to support neurite outgrowth after 1 day of co-culture [126]. Interestingly, to date, nanotopographical features have been shown to play a more important role in the interaction between astrocytes and neurons. Here, a particular roughness of 32 nm has been shown to affect the interaction between those two cell types by making neurons more likely to grow on a rough surface than astrocytes. Moreover, in the case of both lower (3.5–16 nm) and higher (44–70) roughness of surface neurons, surface neurons ultimately preferred to grow on astrocytes instead of the surface. Moreover, neurons growing on a substrate with a roughness of 32 nm were characterized by a significantly lower number of neurites. Nevertheless, those neurites were significantly longer than with any substrate with roughness between 3.5 and 70 nm. Finally, neurons growing on substrate characterized by a roughness of 32 nm were characterized by significantly higher calcium efflux and acetylcholinesterase activity. In the same study, the authors showed that in the case of neural stem cell differentiation, particular roughness (16 and 32 nm) of the substrate might take over biological signals directing NSCs into astrocytes and make them differentiate into neuronal lineages [127]. Neurite outgrowth has also been altered by the nanotopography of electrospun poly-l-lactic acid fibres. Whole DRGs have been shown to produce significantly shorter and thinner neurites when cultured on dedicated and pitted fibres compared with smooth fibres. Nevertheless, this trend was reduced when fibres were coated with laminin. Dissociated neurons were not able to grow on fibres that were not coated with laminin. Subjected to laminin-coated fibres, total neurite length has been increased for neurons cultured not on smooth but on dedicated and pitted fibres, while more branching points between neurites, have been observed. Such findings robustly show that proper use of nanotopography material should be considered when considering a different form of cell culture for obtaining satisfying neuronal growth [128]. As mentioned above, surface roughness may also affect neuritis branching, associated with signal processing between neurons. Since the nervous system consists of a very complex network of neuronal cells, the designed impact on this connectivity is significant for neural tissue regeneration. Onesto et al. recently used real-time calcium imaging and information theory that nanoroughness is a key factor affecting information processing between primary neurons. In the roughness range between 0 and 30 nm, higher roughness has been associated with increased activity of neurons and increased clustering coefficient, small-worldness, simulated information, and decreased characteristic path length in neural networks [129]. These results show how important nanotopography is for neuronal regeneration and restoration of the proper functioning of nervous tissue. On the other hand, surface topography may directly improve neuronal differentiation of neuroblasts and neural stem cells. As cluster-assembled zirconia surfaces of designed nanoroughness corresponding to the extracellular matrix, nanotopography has been shown to promote neuronal differentiation of the murine PC-12 neuroblastic cell line and the maturation of rat hippocampal neurons. In the case of PC-12 cells cultured on nanostructured zirconia (ns-Zr15) substrates, it has been shown that such substrates promote neuronal differentiation by affecting cytoskeletal organization and focal adhesions. PC-12 cells cultured on ns-Zr15 substrates were characterized by increased p-CREB expression. More significantly, ns-Zr15 and ns-Zr25 substrates have been shown to enhance not only neurite outgrowth in neuronal cells but also their electrophysiological activity [130, 131]. In contrast, the enhanced conductivity of materials designed for neuronal cell culture has also been shown to offer an advantage in neuronal differentiation. Electrospun carbonized cellulose scaffolds are characterized by 10^8^ higher electrical conductivity than unfunctionalized electrospun cellulose. Accordingly, scaffolds have been promoting neuronal differentiation and the formation of cellular connections in SH-SY5Y cells, proving that proper conductive properties of the material can be another useful parameter for successful neuronal differentiation enhancement to modulate the properties of scaffolds [132].

## Conclusions

This paper reviews the significance of physical interactions and mechanotransduction in homeostasis and pathology of the nervous system. Recent evidence indicates that changes in nervous tissue mechanics affect the neuroinflammation process, axonal outgrowth, and neuronal differentiation. This knowledge opens the prospect of a vast application potential. Understanding how the mechanical properties of tissue, mechanotransductive and mechanosensitive protein expression, and nanotopography change during disease progression allows the creation of more reliable experimental systems, including hydrogels and spheroid cultures, and designed scaffolds. Moreover, these changes may be traced by both noninvasive methods such as magnetic resonance elastography, and more invasive procedures such as a mechanical characterization of biopaths. In particular, recent evidence indicates that such changes occur at very early stages of disease progression. Finally, drugs can be designed that act on particular mechanosensitive components whose function is affected by pathological conditions. Nevertheless, many gaps exist in the understanding of the role of mechanical factors in neurological disease progression. A better understanding of particular mechanosensitive proteins’ roles in particular disease progressions is fundamental to considering them as targets for new therapeutic approaches. The development and optimization of physical methods for the characterization of the mechanical properties of brain tissue is another significant challenge. The development of less invasive methods is emerging, while the creation of highly repetitive assays based on indentation and rheological methods has to be considered before successfully implementing mechanical testing in routine clinical practice. Interdisciplinary studies among clinicians, biologists, physicists, chemists, nanotechnologists, and specialists from related fields are extremely important for creating a developing environment for further research in this field.

## Abbreviations

AD: Alzheimer’s disease
AFM: Atomic force microscopy
CNS: Central nervous system
CSPGs: Chondroitin sulphate proteoglycans
CIL: Contact inhibition of locomotion
ECM: Extracellular matrix
FAK: Focal adhesion kinases
GFAP: Glial fibrillary acidic protein
MRE: Magnetic resonance elastography
MSC: Mesenchymal stem cells
MAP-2: Microtubule-associated protein 2
MCAO: Middle cerebral artery occlusion
NSCs: Neural stem cells
PDMS: Polydimethylsiloxane
ROS: Reactive oxygen species
ROCK: Rho-associated protein kinase
TFM: Traction force microscopy
TBI: Traumatic brain injury
